# Sphingosine 1-Phosphate in Malaria Pathogenesis and Its Implication in Therapeutic Opportunities

**DOI:** 10.3389/fcimb.2020.00353

**Published:** 2020-08-14

**Authors:** Gunanidhi Dhangadamajhi, Shailja Singh

**Affiliations:** ^1^Department of Biotechnology, North Orissa University, Baripada, India; ^2^Special Centre for Molecular Medicine, Jawaharlal Nehru University, New Delhi, India

**Keywords:** sphingosine 1-phosphate, malaria, RBC, rosette, therapeutic

## Abstract

Sphingosine 1-Phosphate (S1P) is a bioactive lipid intermediate in the sphingolipid metabolism, which exist in two pools, intracellular and extracellular, and each pool has a different function. The circulating extracellular pool, specifically the plasma S1P is shown to be important in regulating various physiological processes related to malaria pathogenesis in recent years. Although blood cells (red blood cells and platelets), vascular endothelial cells and hepatocytes are considered as the important sources of plasma S1P, their extent of contribution is still debated. The red blood cells (RBCs) and platelets serve as a major repository of intracellular S1P due to lack, or low activity of S1P degrading enzymes, however, contribution of platelets toward maintaining plasma S1P is shown negligible under normal condition. Substantial evidences suggest platelets loss during *falciparum* infection as a contributing factor for severe malaria. However, platelets function as a source for plasma S1P in malaria needs to be examined experimentally. RBC being the preferential site for parasite seclusion, and having the ability of trans-cellular S1P transportation to EC upon tight cell-cell contact, might play critical role in differential S1P distribution and parasite growth. In the present review, we have summarized the significance of both the S1P pools in the context of malaria, and how the RBC content of S1P can be channelized in better ways for its possible implication in therapeutic opportunities to control malaria.

## Introduction

Chemotherapeutics is the mainstay of malaria control in absence of high efficacy approved vaccine and ineffective vector control measures (Tibon et al., 2020). Unfortunately, parasite resistance to almost all the current pharmacotherapy is a major threat to control malaria. Despite high incidence, luckily, only a minority of individuals (1–2%) develop the malaria attributed complicacy. However, notable numbers (15–20%) of these patients still succumb to death even after effective antimalarial treatment (Dondorp et al., 2010; Newton et al., 2013) and thus requires the most urgent attention and intensive care. The complex interactions between the malaria parasite and human host leading to adhesion phenotypes (such as cyto-adherence, rosetting, and platelet mediated clumping), endothelial dysfunction, deregulation of the homeostasis system, host inflammatory response and nitric oxide (NO) production are thought to be the key events (van der Heyde et al., 2006; Yeo et al., 2008; Rowe et al., 2009). In other words, host response to malaria infection is of paramount importance in determining inter-individual variation in disease severity in malaria. Host response in malaria research has primarily focused on protein component and lipids were usually appraised for their role in cell membrane formation or energy storage. Recently, Sphingosine 1-Phosphate (S1P), a pleiotropic lysophospholipid has been shown to affect various cellular pathways including the immune and vascular systems which are implicated in the pathology of severe malaria (Snider et al., 2010; Lou et al., 2011; Xiong et al., 2014). S1P is present in almost all cells and body fluids. However, it is one of the important components of blood, distributed differentially in blood cells and plasma, thereby regulating various physiological processes. In the present review, attempt has been made to highlight the current knowledge on S1P metabolism, cellular sources of plasma S1P, their transport and biological function based on the available literatures. In addition to this, we compiled the importance of S1P in the context of clinical malaria. Besides, RBC being the major source of S1P and preferential site for parasite seclusion, how RBC content of S1P could be channelized in a beneficial way for possible malaria control have been discussed.

## S1P Metabolism and Plasma Source of S1P

Shingosine, named after Sphinx, a Greek mythical creature of enigmatic activity is one of the important intermediate in sphingolipid metabolism. S1P is produced mostly through intracellular phosphorylation of sphingosine, a deacylated product of ceramide in various cell types by two isoform of sphingosine kinases (SPHK1 and SPHK2) (Ksiazek et al., 2015; Hatoum et al., 2017). Ceramide in turn is generated either from the condensation of serine and palmitoyl-CoA in a *de novo* pathway by serine palmitoyltransferase in the endoplasmic reticulum (ER), or from degradation of membrane sphingolipids (such as sphingomyelin and glycosphingolipids) in lysosomes or in the outer leaf-let of cellular membranes (Maceyka and Spiegel, 2014; Thuy et al., 2014; Ksiazek et al., 2015; Cantalupo and Di Lorenzo, 2016). The SPHK1 and SPHK2 kinases which phosphorylate sphingosine into S1P have different sub-cellular localization and tissue distribution (Maceyka et al., 2005). While SPHK1 is localized in cytosol (Pitson et al., 2005; Chan and Pitson, 2013) and highly substrate specific (Ksiazek et al., 2015), SPHK2 is mainly found in the nucleus, mitochondria, and ER (Cantalupo and Di Lorenzo, 2016) with broader range substrate specificity (Venkataraman et al., 2006; Maceyka and Spiegel, 2014). Further, of the two isoforms, only SPHK1 is shown to be released out of the endothelial cells constitutively, and the rate of extracellular S1P synthesis by SPHK1 is dependent on the level of spingosine present in the extracellular medium (Ancellin et al., 2002; Venkataraman et al., 2006; Takabe et al., 2008). However, its contribution as an additional source of plasma S1P in human is not clearly known. Moreover, it remains to be seen whether malaria infection has any impact on endothelial cell release of SPHK1 in extracellular milieu. It is established that S1P level in plasma and lymph is maintained relatively high compared to its concentration in tissues (Schwab et al., 2005; Peest et al., 2008; Takabe et al., 2008). Further, this differential gradient of S1P has been shown important in regulating various physiological function (Kerage et al., 2014; Cantalupo and Di Lorenzo, 2016; Cartier and Hla, 2019).

**Figure 1:**
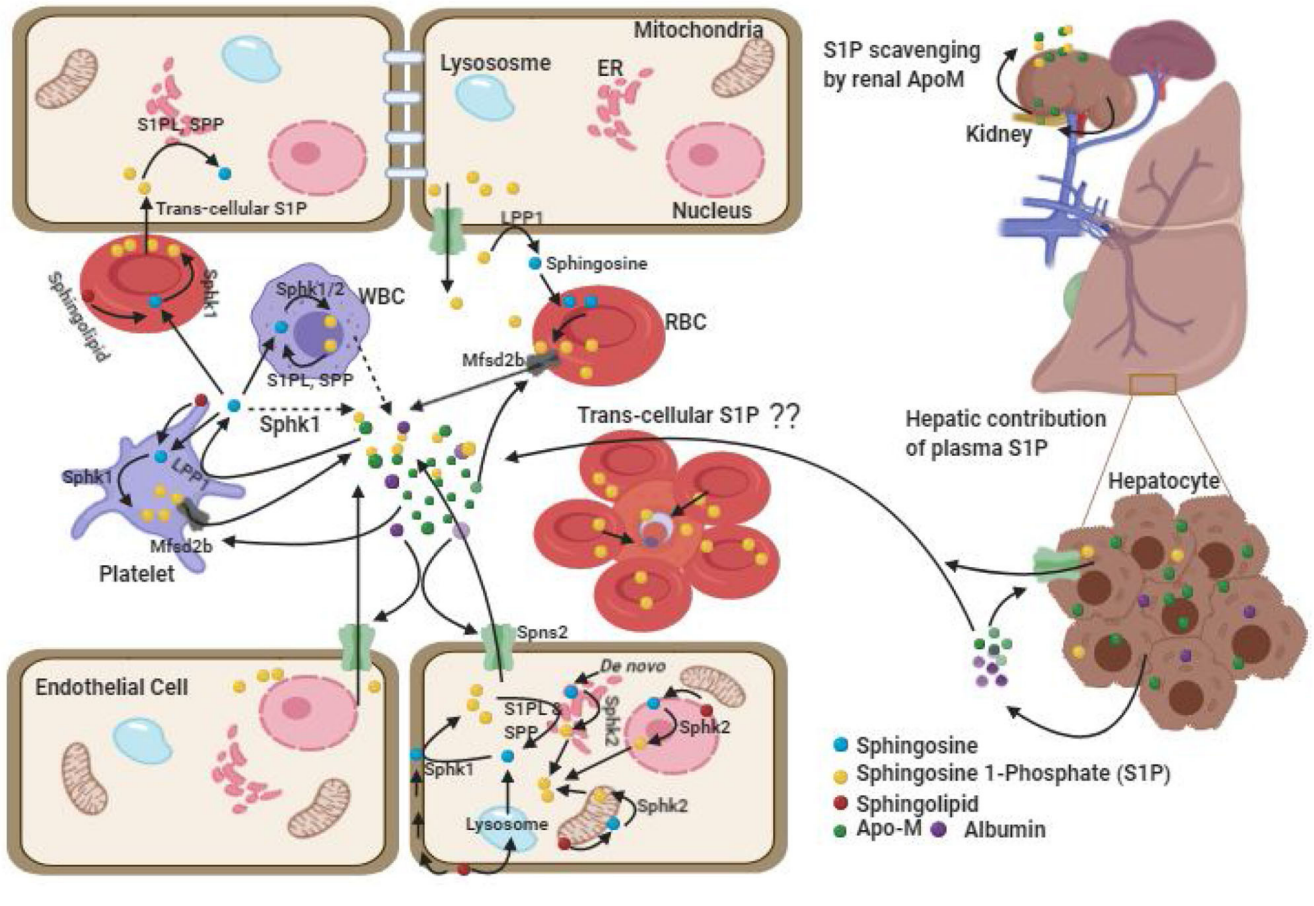
Plasma Sources of cellular S1P. There are two pools of S1P, intracellular and extracellular each with different functions. The intracellular pool is synthesized upon incorporation of the substrate from plasma or its generation within the cell followed by phosphorylation by resident kinases such as SPHK1 in cytosol and SPHK2 in mitochondria, ER and nucleus. The intracellular S1P can be degraded by S1PL and SPP enzymes. The extra-cellular pool of S1P is predominantly maintained by cellular efflux of S1P facilitated by the acceptor molecules (ApoM-HDL and albumin) and possibly by endothelial cell secretion of SPHK1 which converts sphingosine in extracellular space into S1P (dashed arrow). RBCs play important role by functioning as a major reservoir for intracellular and extra-cellular S1P. In absence of acceptor molecule, RBC transports the S1P to EC and other cells through trans-cellular transportation after tight cell-cell contact. While, ECs have been demonstrated as alternative source for plasma S1P, platelets contributes to the extracellular pool only upon activation. Hepatocytes by secreting ApoM stimulate S1P export from hepatic and extra-hepatic cells, whereas kidney derived ApoM function to prevent urinal secretion of S1P and is metabolized in the kidney itself. The (??) in the figure depicts possible trans-cellular transportation of S1P from un-infected RBC to infected RBC in rosette.

It is to be noted that blood harbors S1P in blood cells as well as in plasma (Figure 1). However, plasma S1P has a short half-life period (Venkataraman et al., 2008; Salous et al., 2013) which indicates its rapid clearance and instant replenishment in a well-regulated process. Although blood cells such as RBC and platelets, vascular endothelial cell, and hepatocytes are considered as the important sources of plasma S1P, their extent of contribution is still debated (Tani et al., 2007; Venkataraman et al., 2008). The balance between synthesis and export of S1P, and its intracellular or extracellular degradation by three types of enzymes is crucial in maintaining plasma level S1P. The intracellular degradation of S1P is carried out by two endoplasmic reticulum localized enzymes, such as (i) S1P phosphatases (SPP), which reversibly dephosphorylate S1P into sphingosine and (ii) S1P lyases (S1PL), which irreversibly inactivate S1P in to hexadecenal and ethanolamaine-1-phosphate (Bandhuvula and Saba, 2007; Liu et al., 2012). The degradation of S1P in extracellular space is carried out by the ecto-enzyme lipid phosphate phosphatases (LPP1 and LPP3) found in the plasma membrane of endothelial cell, and platelets (Yatomi et al., 2004; Zhao et al., 2007; Salous et al., 2013), which dephosphorylates most of the lipid phosphates including S1P. The S1P in the cell can also be dephosphorylated by another isoforms of LPP, the LPP2 which resides intra-cellularly (Kai et al., 2006). Of note, circulating S1P is rapidly dephosphorylated by LPPs into sphingosine (Zhao et al., 2007), where the latter is taken up by the blood cells and platelets (Tani et al., 2005; Ksiazek et al., 2015) for intracellular synthesis of S1P (Zhao et al., 2007). Besides, sphingosine can also be generated from membrane sphingolipids in these cells.

Of the various cell types, RBC synthesize S1P mainly via SPHK1, whereas platelets synthesize via SPHK2 and they both accumulate large amount of S1P due to lack of cellular organelles and S1P degrading enzymes (Hanel et al., 2007; Zhao et al., 2007; Bode et al., 2010; Urtz et al., 2015). Although the platelet content of S1P is relatively higher than that is in the RBC (Anada et al., 2007; Ito et al., 2007), the majority of S1P in the plasma pool of healthy individuals is contributed by RBC, possibly due to its largest share in total blood cell numbers (Hanel et al., 2007; Ito et al., 2007) and that the platelets release S1P only upon activation (Ohkawa et al., 2008; Ono et al., 2013). It is shown that human platelets can also constitutively release S1P without stimulation depending upon the presence of high albumin concentration in the plasma (Jonnalagadda et al., 2014). However, the observation of normal level of plasma S1P in thrombocytopenic or platelet-deficient mice (Pappu et al., 2007; Venkataraman et al., 2008) and non-correlation of platelet count with S1P level in human study (Ohkawa et al., 2008) suggest the contribution of platelet toward maintaining plasma S1P is negligible under normal condition. Interestingly, while genetic and pharmacological inhibition of *Sphk1* has shown reduction in plasma level of S1P by 50% (Kharel et al., 2011), similar studies with *Sphk2* (which encodes the major enzyme for platelet S1P synthesis) showed opposite results (Sensken et al., 2010; Kharel et al., 2012). This also supports the non-significant role of platelets toward plasma S1P. In humans, although the cellular content of S1P in RBC of both anemic and healthy individuals is found to be comparable (Bode et al., 2010; Selim et al., 2011), significantly reduced plasma S1P level in anemic patients suggests the major role of RBC in contributing S1P in human blood (Hanel et al., 2007; Bode et al., 2010; Selim et al., 2011). This was further strengthened by the observation that in *Sphk1/Sphk2* deficient mice, transfusion of only wild type erythrocytes but not the platelets and leukocytes restored normal S1P level in plasma (Pappu et al., 2007). Further, significant correlation of RBC parameters with plasma S1P and the findings of higher plasma S1P in men, compared to women (Ohkawa et al., 2008) highlight RBC as the important source of blood S1P. Although gender difference in plasma S1P level was shown controversial in other studies (Hammad et al., 2010; Karuna et al., 2011), it is attributed to be due to remarkable decrease in plasma S1P post-menopause in women because of estrogen deficiency (Guo et al., 2014) and the inclusion of older age group in the study (Guo et al., 2014).

Spontaneous S1P efflux from vascular endothelial cells have also been documented similar to RBCs and could serve as an alternative source in replenishing plasma S1P at least in mice (Venkataraman et al., 2008; Fukuhara et al., 2012; Hisano et al., 2012). Shear stress and down regulation of intracellular S1P degrading enzymes have been shown to enhance S1P efflux from ECs (Venkataraman et al., 2008). However, it could not be replicated in human studies (Zhao et al., 2007). The contribution of WBCs toward plasma S1P is almost none and suggested to be due to its high rate of degradation than the rate of its release (Hanel et al., 2007; Salous et al., 2013). Consistent to this, no correlation between WBCs count with S1P has been found (Pappu et al., 2007; Ohkawa et al., 2008). The role of hepatocytes in S1P homeostasis is related to its over expression of ApoM (Kurano et al., 2013; Nojiri et al., 2014), the major acceptor of circulating S1P which stimulates S1P export from hepatic and extra-hepatic cells (Liu et al., 2014; Huang et al., 2015). Although liver and kidney cells secrete ApoM (Zhang et al., 2003; Huang et al., 2015), plasma level of ApoM which mainly associates with HDL is predominantly secreted by liver cells. Liver derived ApoM play a major role in sphingolipid recycling, and in the maintenance of the plasma level of ApoM/S1P-enriched HDL besides whole body S1P distribution and homeostasis (Venkataraman et al., 2008; Christoffersen et al., 2011; Kurano et al., 2013). However, renal-ApoM by functioning as an S1P scavenger is metabolized in the kidney itself after re-absorption in the renal proximal tubule through the megalin receptor and prevent urinal secretion of S1P (Faber et al., 2006). Altogether, RBC is the major source of plasma S1P in humans but vascular EC and RBCs are equally important in maintaining blood S1P in mice.

## Export of Intracellular S1P, Acceptor Molecules and Their Effect on S1P Function

Having a polar head group, the export of cellular S1P to plasma is carried out through transporters like spinster homolog 2 (Spns2) from endothelial cells in a non-ATP dependent manner (Fukuhara et al., 2012; Hisano et al., 2012; Mendoza et al., 2012; Donoviel et al., 2015) or through major facilitator superfamily transporter 2b (Mfsd2b) from RBCs and platelets in an ATP dependent manner (Vu et al., 2017; Kobayashi et al., 2018). Moreover, being poorly water soluble, the release of S1P to plasma is facilitated by the presence of extracellular acceptor molecules such as HDL (most notably ApoM-HDL which carries 70% of S1P), or albumin (which carries 30% of S1P). Intracellular S1P efflux to plasma has been decreased upon dilution of plasma or prevented in serum/plasma free medium (Hanel et al., 2007; Venkataraman et al., 2008; Bode et al., 2010; Christoffersen et al., 2011; Karuna et al., 2011; Sutter et al., 2014). Further, in absence of the S1P acceptor molecules (albumin and ApoM) in plasma, RBC may contribute to S1PL degradable cellular pool of S1P through trans-cellular S1P transportation to EC and other tissue cells upon tight cell-cell contact (Bode et al., 2010). This indicates that RBC content of S1P and the acceptor molecules play critical role in differential distribution and degradation of S1P.

Recent studies have shown that the S1P efflux from EC, RBC, and platelets, and their effective circulation in the plasma and specific biological function is dependent on its association with the type of acceptor molecule. Of note, the rate of release of S1P from RBC is enhanced by the ApoM-HDL (Bode et al., 2010; Wilkerson et al., 2012; Yu et al., 2014) and from the platelets by albumin (Jonnalagadda et al., 2014). Further, functional differences in evoking intracellular signaling between ApoM-S1P and albumin-S1P exist (Wilkerson et al., 2012; Galvani et al., 2015; Obinata and Hla, 2019), albeit the reason for this remains poorly understood. It is suggested that while albumin is indiscriminating in binding to various small hydrophobic molecules including S1P (Obinata and Hla, 2019), ApoM has high affinity for S1P and is a specific chaperone that protects the bound S1P from degradation by LPP (Kimura et al., 2001; Christoffersen et al., 2011; Zhang et al., 2016). Moreover, ApoM-bound S1P in a context-dependent signaling has been shown to mediate prolonged receptor activation for several hours compared to transient activation by albumin in evoking downstream cascade (Wilkerson et al., 2012; Swendeman et al., 2017). While albumin-bound S1P has been found to be associated with diminished cAMP production and efficient S1PR_1_-internalization (Galvani et al., 2015; Obinata and Hla, 2019), ApoM-S1P has been observed to regulate lymphopoiesis in bone marrow (Blaho et al., 2015) and maintain vascular integrity through anti-inflammatory responses in endothelial cells (Galvani et al., 2015; Sattler et al., 2015; Keul et al., 2019). Interestingly, engineered ApoM with long plasma half-life (about 90 h) and the potential to bind S1P (such as ApoM-Fc) was when intraperitoneally administered to the experimental model of stroke in mice, an increase in plasma S1P concentration by 30% was recorded post 24 h with protection from myocardial damage after ischemia, together with reduction of hypertension and brain infarct volume compared to the control ApoM of comparable half-life with no S1P binding ability (ApoM-Fc-TM) (Swendeman et al., 2017). Moreover, the association of low level HDL-S1P content with coronary artery disease (Sattler et al., 2010), and type II diabetes (Tong et al., 2014) in case-control human studies evidence for the potential beneficial role of ApoM-S1P in these diseases. However, it remains to be elucidated how plasma S1P level and the S1P bound receptors affect the pathological conditions in human malaria.

Although intracellular receptor for S1P is not well-characterized, it is shown to regulate important intracellular function like ubiquitination, histone deacetylation and gene expression, and respiration (Hait et al., 2009; Alvarez et al., 2010; Sun et al., 2016). On the other hand, the extracellular S1P exerts its diverse effects in an autocrine or paracrine manner through five G-protein coupled receptors [S1PR_1−5_, initially termed as endothelial differentiation gene (EDG)]. These S1PRs vary in their distribution and expression in response to various stimuli (Strub et al., 2010; Blaho and Hla, 2014). Of the five receptors (S1PR_1−5_), S1PR_4−5_ have much narrower patterns of expression compared to S1PR_1−3_ receptors, with ubiquitous expression of S1PR_1_ in the brain, kidney, lung, spleen and cardiovascular system (Strub et al., 2010; Blaho and Hla, 2014). While S1PR_1_ regulates immune cell function including lymphocyte egress from lymph nodes (Liu et al., 2000; Schwab et al., 2005; Sanna et al., 2006), S1PR_2_ is required for vestibular and auditory systems development (MacLennan et al., 2006; Ingham et al., 2016; Hofrichter et al., 2018), whereas S1PR_3_ is required for regulation of heart rate (Forrest et al., 2004). Further, S1PR_1−3_ receptors play an important role in angiogenesis and vascular permeability (Strub et al., 2010). A study involving triple knockout of S1PR_1−3_ has led to the embryonic lethality in mice due to the massive vascular deficiencies (Kono et al., 2004). Since, vascular injury is common in severe *falciparum* malaria; we hypothesize for the possible involvement of S1PR_1−3_ receptors including plasma level of S1P in severe malaria. Although the S1P concentration in plasma and its subsequent effect on malaria could possibly be due to genetic variation, there is lack of information on mutations in genes affecting S1P synthesis (*Sphk1, Sphk2*), degradation (*Spl, Spp1, Spp2*) and signaling receptors (*S1pr*_1−3_) leading to plasma level of S1P concentration and clinical outcome in malaria. Screening of functional genetic variations in these genes may be useful for better understanding of S1P biology in malaria.

## S1P and Its Association with Malaria

To date, only few studies have exploited the role of S1P in malaria. Recent studies have highlighted the dysregulation of the S1P pathway mostly in the pathogenesis of CM in mice (Table 1). Reduced plasma S1P has been found to be associated with malaria severity and impaired neuro-cognitive functions in mice (Table 1). In Ugandan children with CM, the plasma S1P level was significantly less compared to uncomplicated malaria (Finney et al., 2011) consistent to the results obtained in experimental malaria. Severe neurological sequelae are also common in human CM, often associated with death in children post**-**discharge from hospital (Idro et al., 2010; Oluwayemi et al., 2013). Of note, increased bio-availability of S1P has been shown to inhibit neurological signs and prolonged survival in experimental CM (Finney et al., 2011; Nacer et al., 2012, 2014) indicating S1P enrichment in plasma could be a rational means of new therapeutics strategy, although it has to be established in human studies. Further, the involvement of S1P receptors, specifically increased S1PR_3_ concentration with mortality has been documented in acute lung injury (ALI)/acute respiratory distress syndrome (ARDS) patients (Sun et al., 2012), yet another severe complication of *falciparum* malaria which ranges from 5 to 25% of adults and 29% of pregnant women (Taylor et al., 2012). Anticipating implication of circulating S1PR_3_ as a potential biomarker of severity in malaria induced ARDS, when the expression of the *Sphk1* and S1PR_3_ proteins were investigated in malaria-associated ALI/ARDS, up-regulated expression of these were demonstrated in the lung tissues of experimental mice and human malaria patients (Punsawad and Viriyavejakul, 2019; Viriyavejakul and Punsawad, 2020). However, agreeing to evidence from previous studies (Finney et al., 2011; Punsawad and Viriyavejakul, 2017), plasma and lung tissues level of S1P was significantly less in malaria infected mice with ALI/ARDS) compared to non-ALI/ARDS and control (Punsawad and Viriyavejakul, 2019). Although the reason for S1P reduction remains unknown, it can be surmised that *Sphk1* and *S1pr*_3_ expression in lung tissues do not contribute much for tissue fluids or plasma level of S1P. A recent knockout study in *Pseudomonas aeruginosa* induced lung inflammation revealing the pathogenic role of SPHK2 through epigenetic regulation of gene expression and intracellular S1P generation (Ebenezer et al., 2019) proposes the possible collusion of SPHK2 in malaria induced ARDS. Further studies with *Sphk1, Sphk2*, and *S1pr*_3_ knockout mice or antagonists may help understanding their involvement in ALI/ARDS and unveil the reason for S1P reduction. These findings are of particular interest and indicate the possible protective nature of S1P in severe malaria; however the role of S1P in malaria is still unclear.

**Table 1:** Association of S1P with experimental infection of malaria and clinical malaria in human.

**S. No**	**Organism or cell**	**Experimentation**	**Findings**	**References**
1	Human Erythrocytes	The *SPHK-1* activity and its phosphorylation status were compared between uninfected and *P*. *falciparum*-infected erythrocytes *in vitro* in different stages of asexual development.	A significant decrease in SPHK-1 phosphorylation and activity were observed in a time-dependent manner in *falciparum*-infected erythrocytes compared to uninfected RBCs.	(Sah et al., 2020)
2	Human Erythrocytes	Intracellular parasitic growth was assessed upon pharmacological inhibition of host cell *SPHK1* in *falciparum* infected RBCs	Intracellular reduction of S1P in erythrocytes impaired glycolysis, low level of lactate was released as bi-product; parasite growth was affected leading to cell death. Impaired glycolysis was attributed to be due to decreased translocation of GAPDH from membrane to site of function in cytosol in infected RBC.	(Sah et al., 2019)
3	DBA/2 mice	*Plasmodium berghei* ANKA infected mice were divided into two groups: with and without ALI/ARDS. Expression of the *SphK-1* and S1PR-3 proteins was investigated	Upregulated expression of the *SphK-1* and S1PR-3 proteins was observed in endothelial cells, alveolar epithelial cells and alveolar macrophages in the lung tissues of ALI/ARDS group compared to control group. However, S1P in plasma and lung tissues was significantly less in ALI/ARDS group compared to control mice.	(Punsawad and Viriyavejakul, 2019)
4	HUVECs	Endothelial cells were incubated with malaria infected and non-infected human sera for induction of permeability. Treatment with FTY720 before and after incubation of sera was evaluated for restoration of permeability.	Significantly high permeability was recorded after incubation with serum from complicated patients with *falciparum* malaria compared to uncomplicated *falciparum* patients and *vivax* infected patients. Treatment with FTY720 before and after incubation of sera significantly reduced and prevented sera induced increase in endothelial cell permeability	(Oggungwan et al., 2018)
5	CBA/CaJ mice	Mice were infected with *PbA* for induction of ECM and with *Plasmodium yoelii* for malarial hyperparasitemia without neurological impairment. Treatment with FTY720 was investigated to understand the pathogenesis	Recruitment of activated leukocytes (CD8+ T cells and ICAM+ macrophages), and neutrophils to post-capillary venules prevents venous blood efflux from the brains severely, which leads to vasogenic edema in ECM. Cells arrest in vasculature is likely to increase the intracranial pressure leading to poor clinical outcome. Treatment with FTY720 prevents these cells recruitment and protect from death in ECM.	(Nacer et al., 2014)
6	CBA/CaJ mice	Mice infected with *P. berghei ANKA* strains were evaluated for recovery upon FTY720 treatment (oral dose of 0.3 mg/kg/day, starting 1 day before infection)	*P. berghei* ANKA induced ECM with neurological signs, cerebral hemorrhages, and BBB dysfunction. FTY720 inhibited vascular leakage and neurological signs, stabilized BBB and prolonged survival to ECM.	(Nacer et al., 2012)
7	C57BL/6	Mice infected with *PbA* (ECM) were treated with FTY720 1 d prior to and 1, 3, or 5 d post-infection to evaluate the possible involvement of S1P in endothelial dysfunction and survival. In order to examine whether increased S1P bioavailability affect survival in ECM, parasite infected mice deficient of S1PL was compared with wild-type littermates	FTY720 treatment in 1 day–pre-infection and 1 day–post-infection improved BBB integrity in ECM compared to untreated infected mice, and day 3 post-infection treated mice. Lymphocyte counts were markedly reduced in blood and their infiltration (both CD4^+^and CD8^+^ cells) in the brain was decreased. Further, treatment with FTY720 suppressed endothelial dysfunction and reduced plasma level of IFN⋎. FTY720 in combination with sub-curative dose of artesunate treatment on 5d post-infection resulted in improved survival compared to artesunate therapy alone. Increased bioavailability of S1P due to S1PL deficiency in knockout mice was associated with improved outcome compared to wild-type littermates	(Finney et al., 2011)
8	Human	Plasma S1P levels was compared between *falciparum* infected Ugandan children with CM and UM to examine the significance of S1P in human malaria	Plasma level of S1P was significantly less in children with cerebral malaria compared to UM. Also hemoglobin and platelet levels were highly reduced in CM	(Finney et al., 2011)
9	Human	S1P levels in serum of adult Indian malaria patients (*falciparum* and *vivax* infected) were compared to healthy controls	S1P level was significantly less in malaria patients compared to healthy control. Complicated malaria patients had the lowest S1P level, and platelet count positively correlated with S1P level	(Sah et al., 2020)
	Human	Autopsied patients who died with *P. falciparum* malaria were categorized into PE and Non-PE group, and cellular expression of SPHK-1 and S1PR-3 were investigated using immune histochemistry.	Over expression of both SPHK-1 and S1PR-3 proteins were observed in lung tissues of PE indicating their role in the pathogenesis of pulmonary complications in severe malaria.	(Viriyavejakul and Punsawad, 2020)
10	Human	Endothelial glycocalyx breakdown products, markers of endothelial dysfunction, parasite biomass, S1P and NO levels were compared between Indonesian adults with *falciparum* malaria (severe and moderately severe) and healthy control	Inverse correlation between S1P and breakdown products of endothelial glycocalyx was observed. Glycocalyx breakdown was associated with endothelial dysfunction, low NO bioavailability, increased parasite biomass, severity of malaria and risk of death.	(Yeo et al., 2019)
11	Human	Serum S1P levels were measured in Thai patients with *P*. *vivax*, uncomplicated *P*. *falciparum*, and complicated *P*. *falciparum* malaria on day 0 and on day 7. These values were compared to that of healthy control	Low serum S1P was associated with severity of malaria on day of admission which increased to significant level on day 7. Platelet count, hemoglobin and hematocrit values were positively correlated with serum S1P level in severe *falciparum* malaria patients	(Punsawad and Viriyavejakul, 2017)

*GAPDH, Glyceraldehyde 3-phosphate dehydrogenase; SPHK1, Sphingosine kinase 1; ALI/ARDS, acute lung injury/acute respiratory distress syndrome; HUVEC, Human umbilical vein endothelial cell; ECM, Experimental cerebral malaria; BBB, Blood-brain-barrier; UM, Uncomplicated malaria; PE, Pulmonary edema; S1PL, Sphingosine-1-phosphate lyase*.

Endothelial dysfunction leading to loss of vascular integrity and leakage is the key to the pathogenesis of severe malaria (van der Heyde et al., 2006; Liles and Kain, 2014). Accelerated breakdown of endothelial glycocalyx leading to endothelial cell (EC) activation and exposure of EC surface receptors for parasite sequestration has been documented during severe malaria (Hempel et al., 2019; Yeo et al., 2019). Interestingly, inverse association of S1P with endothelial glycocalyx breakdown products have been demonstrated in Indonesian adults patients with *falciparum* infection (Yeo et al., 2019). Further, increased permeability of endothelial cells induced by sera from individuals with complicated *falciparum* malaria has been found to reverse with phosphorylated FTY720, an S1P agonist (Oggungwan et al., 2018). This indicates that S1P bioavailability could be instrumental in attenuating endothelial damage and ensuing protection from severe malaria. Previous study examining the impact of S1P bioavailability through S1P lyase deficiency in ECM has documented improved survival in mice (Finney et al., 2011), which further resolves the importance of S1P in effective therapeutics against malaria. Disruption of vascular integrity in the blood–brain barrier (BBB) is often associated with poor outcome in CM. Moreover, increased vascular integrity and significant survival advantage have been observed in murine model of CM upon prophylactic or early therapeutic treatment with FTY720. However, therapeutic intervention with FTY720 in later stage infection (3–5 days) in the same study was ineffective against ECM (Finney et al., 2011). Notably, administration of FTY720 in combination with sub curative dose of artesunate (the most potent antimalarial drug against all forms of severe malaria) in late stage infection resulted in improved survival from ECM with unaltered parasite burden compared to artesunate alone (Finney et al., 2011) indicating that the protective effect was especially through host response modulation. Artesunate is shown protective against malaria by its direct parasite killing activity and indirect immune-modulatory effect such as prevention of endothelial cell activation, cyto-adherence of parasite infected RBCs, and amelioration of blood–brain barrier (BBB) breakdown (Souza et al., 2012). Recent study documents artesunate mediated alleviation of BBB disruption to be through activation of S1PR_1_, downstream phosphatidylinositol 3-kinase/AKT signaling, and stabilization of β-catenin (Zuo et al., 2017). Although FTY720 too activates S1PRs (except S1PR_2_) initially, it down regulates S1PR_1_ function through irreversible induction of S1PR_1_ internalization and degradation (Oo et al., 2011). It remains unknown how FTY720 modulates the action of artesunate in improving survival from later stage ECM in combination treatment. Nonetheless, these findings are immensely important to include S1P agonists as potential adjunctive therapeutics for malaria treatment. It is suggested that reduced lymphocyte count in the blood due to impaired lymphocyte egress with significant restriction of immune cells infiltration into the brain upon FTY720 treatment as prophylactic or early therapeutic drug could be the contributing factor for protection against CM (Finney et al., 2011). However, in late stage treatment, when there was already advancement in leukocyte egress and subsequent recruitment to brain had begun (a process involved in CM pathology), attempt to recover mice could have been difficult (Finney et al., 2011; Nacer et al., 2012, 2014). Artesunate has been proven beneficial against late stage ECM independent of parasite killing and is attributed to be due to the reduction of endothelial dysfunction, and leuckocyte detachment from brain vasculature (Clemmer et al., 2011; Souza et al., 2012). Besides, IL-10 (which suppresses inflammation) producing T cells are augmented in artesunate treated mice (Thomé et al., 2016). Although there is rapid decrease in leukocyte accumulation in brain of artesunate treated ECM (Clemmer et al., 2011; Thomé et al., 2016), whether it is through the same mechanism of restricting leucocytes infiltration in to CNS as it for FTY720 is unknown. It is possible that FTY720 by down regulating S1PR_1_ during late stage infection may lead to breach of BBB which may facilitate effective delivery of artesunate to central nervous system (CNS) for execution of immune-protective response against malaria. Some transient pharmacological inhibition studies of S1PR_1_ in brain endothelial cell also highlight enhanced delivery and accumulation of therapeutics drugs in to the CNS (Cannon et al., 2012; Yanagida et al., 2017) supporting this hypothetical dysregulated S1PR_1_ signaling axis as a possible target in modern therapeutics. Besides, S1P induction of NO release from endothelial cell (Igarashi et al., 2001) in mediating protective immune-response cannot be ruled out as the bioavailability of NO has been associated with resistance to malaria in previous clinical studies (Yeo et al., 2008; Dhangadamajhi et al., 2009). However, the effect of S1P and S1PR_1_ signaling in human malaria has not been investigated in terms of NO production and subsequent clinical outcome.

Previous studies investigating the effects of *Plasmodium falciparum* infections on blood indices show reduction of platelet count (thrombocytopenia) and RBCs (anemia), the two major sources for plasma S1P to be frequent and are responsible for clinical severity with fatal outcome (Birhanu et al., 2017; Punsawad and Viriyavejakul, 2017; Dhangadamajhi et al., 2019). Further, platelet count has been delineated to resolve rapidly with recovery in the absence of any additional and specific treatment (Khan et al., 2012). Also, thrombocytopenia and anemia have been associated with a reduction in S1P level (Ono et al., 2013; Punsawad and Viriyavejakul, 2017). Although platelets release S1P into the blood circulation only after activation, whether such activation occurs during *falciparum* infection needs to be investigated. Based on significant correlation between platelet count and plasma S1P (Ono et al., 2013; Punsawad and Viriyavejakul, 2017), and their association with severity of malaria, it is reasonable to speculate the contribution of platelet to plasma S1P in malaria. On the other hand, S1P gradient and S1PR signaling (specifically through S1PR_1_ and partly by S1PR_4_ under stress) were shown essential for platelet formation from megakaryocytes and their shedding in to the circulation (Zhang et al., 2012). Therefore, reduced plasma S1P with impaired S1PR signaling during *falciparum* infection might affect thrombopoiesis and could be the cause of observed thrombocytopenia. Since, platelets harbor endothelial cell protective factors (Nachman and Rafii, 2008), their loss upon reduced S1P in plasma may contribute indirectly to endothelial dysfunction. Whether, platelets function as plasma source of S1P during *falciparum* infection and/or thrombocytopenia in malaria is a consequence of reduced S1P in plasma is yet to be investigated. Although low level of S1P in plasma is associated with cerebral malaria and ARDS (Finney et al., 2011), its concentration in other forms of sub-clinical severe malaria needs further investigation. Besides, the role of S1P in endothelial dysfunction, inflammation, and NO production is largely unknown in human malaria. Therefore, it is imperative to investigate the prognostic implications of S1P in the context of severe *falciparum* malaria for its possible utility as adjunctive therapeutics in severe malaria.

## RBC and Malaria in the Context of S1P

**Figure 2:**
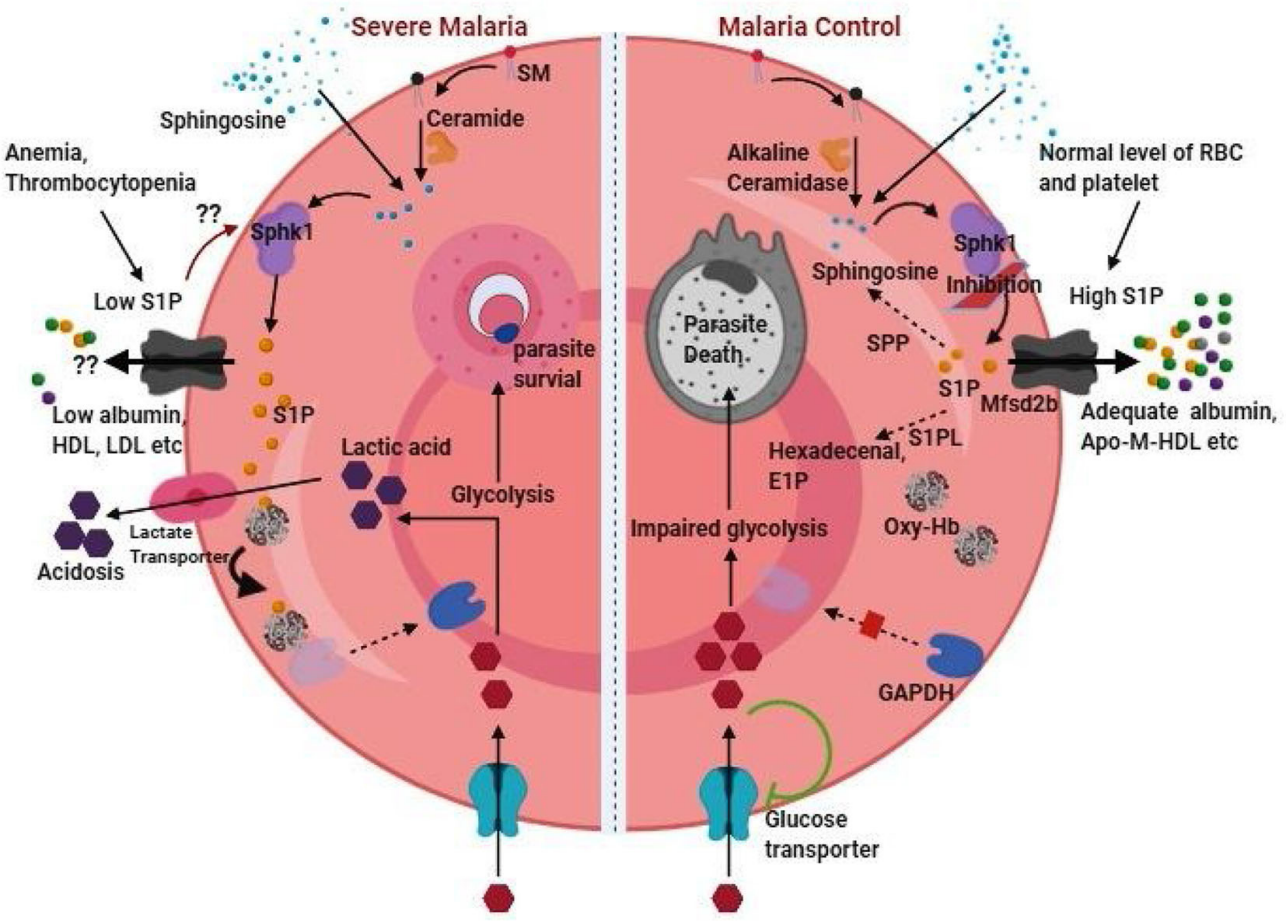
RBC content of S1P as therapeutic target in malaria. RBC content of S1P can function as double-edged sword in malaria pathogenesis by provoking severe malaria (left half of the figure) or can be targeted for malaria control (right half of the figure). Thrombocytopenia and anemia, which are common in malaria indicates loss of two important sources of plasma S1P. Besides, depletion of acceptor molecules such as ApoM and albumin during malaria infection is expected to further harsh the condition of low plasma S1P level by reducing S1P efflux from these cellular sources. On the other hand, low level of plasma S1P might induces SPHK1 for phosphorylation of sphingosine synthesizing S1P in RBC. Sphingosine in RBC can be available as an intermediate of sphingolipid metabolism or directly be incorporated from the plasma. While intra-cellular S1P in RBC facilitate enhanced glycolysis essential for parasite growth, release of lactic acid byproduct leads to the severe condition of acidosis in malaria. Thus, malaria control (right half of the figure) can be achieved through S1P deprivation in parasite infected erythrocyte either by sphk1 inhibition, intracellular degradation by stimulation of S1P phosphatase and lyase, and/or promoting S1P exports in presence of adequate level of acceptor molecules. High plasma S1P has the beneficial role of protection against malaria. The (??) represents our hypothesis based on experimental evidences in different conditions or diseases.

Owing to the major source of S1P in the blood and preferential site for parasite seclusion, the role of RBCs in the context of S1P is critical. In a recent *in vitro* study by our group, it was shown that erythrocytic inhibition of SPHK1 leading to reduced intracellular S1P level arrested parasite growth and ultimately to cell death possibly due to impaired glycolysis (Sah et al., 2019). It was suggested that the deprivation of intra-erythrocytic S1P hindered the binding of deoxy-Hb to plasma membrane thereby decreasing the translocation of the glycolytic enzyme, Glyceraldehyde 3-phosphate dehydrogenase (GAPDH) from the erythrocyte plasma membrane to the cytosol (Sun et al., 2016; Sah et al., 2019). This indicates that besides hemoglobin (major nutrient source for the intra-cellular malaria parasite), intra-erythrocytic level of S1P is important for parasite growth as well, at least for the smooth operation of glycolysis in order to meet its high energy demand. Further, RBC being the main repository for S1P due to its capability to import sphingosine from the plasma (Ito et al., 2007), high SPHK1 activity (Hanel et al., 2007; Pappu et al., 2007), and lack or low activity of S1P degrading enzymes (Ito et al., 2007; Selim et al., 2011) may render survival advantage to the obligatory parasite. In addition, metabolic acidosis, the common cause of severe *falciparum* malaria could be the consequence of S1P mediated glycolysis boost in parasite infected RBC which can lead to increased lactate production. However, the fact that plasma level of S1P is crucial, and its low level has been attributed to cause severe malaria. Although, anemia and thrombocytopenia (which are common during severe malaria) terrify the condition of reduced plasma S1P, there is no study conducted till date addressing whether erythrocytic content of S1P is maintained despite decreased plasma S1P level during severe malaria. Previous study documents RBC content of S1P in non-malaria anemic patients to be similar compared to healthy individual (Selim et al., 2011). Intriguingly, Knapp et al. have documented perpetual reduced plasma S1P concentration as a stimulant for enhanced SPHK1 expression in erythrocytes in myocardial infarction (Knapp et al., 2013). During malaria infection, due to prolonged reduction in plasma S1P, parasite infected and/or non-infected RBCs might also be induced for increased rate of S1P production. Besides, trans-cellular S1P transportation from un-infected RBC to infected RBCs in rosette can't be ruled out. Rosette formation (aggregation of uninfected RBCs around a parasite infected RBC) is shown to be virulent in malaria and is region specific (Rowe et al., 2009; Rout et al., 2012). However, what triggers rosette formation is unknown. It is possible that poor intracellular S1P in parasite infected RBC may favor its binding with un-infected RBCs in rosette for auxiliary S1P to the growing parasite through trans-cellular transportation. In a recent study, the observation of time dependent decrease in SPHK1 activity in infected RBC *in vitro* culture of *falciparum* parasite without affecting normal RBC (Sah et al., 2020) supports a possible acquisition of S1P from nearby uninfected cells. Investigation on this aspect would thus be helpful in better understanding on involvement of S1P in rosette formation and subsequent complicacy in severe malaria. Further, uptake of S1P carrier proteins by the infected erythrocytes during *in vitro* culture (El Tahir et al., 2003) or their significant loss from plasma in malaria infected patients compared to the healthy individual (Visser et al., 2013) suggests strategic reduction of S1P export to the plasma by the parasite during malaria infection. Therefore, we speculate that intra-erythrocytic level of S1P adequate for parasite growth might be maintained during malaria and that decreased S1P efflux to plasma due to insufficient acceptors (Figure 2) may lead to S1P mediated various patho-physiological conditions of malaria.

## Therapeutic Opportunities

Up to now it is clear that Sphk1/S1P signaling nexus and maintenance of adequate S1P level in erythrocytes is essential for intra-cellular parasitic growth (Sah et al., 2019), whereas low plasma S1P concentration contributes to severe manifestation of malaria. Therefore, reduction of RBC content of S1P and/or augmentation of plasma S1P through efflux from cellular sources could be an adjunctive therapeutic opportunities against severe malaria. Since S1P level in body fluids and cellular compartments is tightly regulated by complex interplay of S1P metabolizing enzymes, transporters, chaperones, and signaling receptors, one or more of these could be induced, inhibited or modulated as per requirement for S1P-targeted therapeutic approaches. Amongst the various S1P receptors targeting agents, functional antagonist to S1PR_1_ such as Fingolimod (also known as FTY720-P/Gilenya) and Siponimod (antagonist to S1PR_1_ and S1PR_5_) are FDA–approved drugs (Cartier and Hla, 2019). These drugs are shown to be effective against multiple sclerosis (MS), relapse remitting MS, neuro degenerative diseases, and cancer in several clinical trials (Cartier and Hla, 2019). Further, because of the acute agonistic action upon initial binding Fingolimod has also been reported to induce S1PR_1_ in megakaryocytes resulting in rapid platelet release (Zhang et al., 2012). Keeping in view of the constructive role of FTY720 in maintaining vascular integrity, survival benefits against CM and importance of platelets biology in malaria pathogenesis, FTY720 and related drugs should also be evaluated for their effectiveness in human clinical trial of malaria.

Regulation or inhibition of metabolic enzymes, particularly SPHK1 in RBC and deprivation of RBC content of S1P could be a potent therapeutic against malaria infection. A recent article by Pulkoski-Gross and Obeid (2018) describes SPHK1 regulation at different levels starting from transcription to post-translational modification through the use of long non-coding RNAs, micro RNAs, small interfering RNAs, and physiological and pharmacological stimuli. However, RBC being devoid of nucleus and genetic material, these methods of regulation seem ineffective. On the other hand, requirement of membrane localization for the cytosol resident SPHK1 for its ultimate functioning leave a hope to examine inhibitors of SPHK1 interaction with membranes as possible drug compound. Inhibition of SPHK1 phosphorylation at serine 225 has enabled reduced translocation to membrane resulting into deregulated S1P in *Leishmaniasis* infected macrophages (Arish et al., 2018). Reduced SPHK1 activity in *falciparum* infected RBCs has also been documented due to the lack of phosphorylation (Sah et al., 2020). Recently, GPCR proteins such as bradykinin receptors and muscarinic M3 receptor were shown to induce SPHK1 translocation to membrane (ter Braak et al., 2009; Bruno et al., 2018) and thus their inhibition would be provocative of future research. Further, cellular degradation of SPHK1 leading to loss of S1P in RBCs may also be an alternative approach of restricting intracellular parasite growth. Although on-target inhibition or degradation of SPHK1 is likely to preclude the possibility of trans-cellular S1P transport from uninfected RBCs to parasite infected RBC in rosette, undesirable degradation in other cell types may limit the use of these drugs. Further, this could also prevent participation of RBC as a source to plasma S1P as well hampering the protective effect of S1P against malaria. It is noteworthy that platelets production of S1P is largely mediated by SPHK2 (Urtz et al., 2015). Thus, platelet induction of S1P release could replenish plasma S1P and may play a crucial role in such case. Future studies in this aspect would render additional insights as to the role of RBC and platelets derived S1P in malaria. Of the several compounds, 2-(p-hydroxyanilino)-4-(p-chlorophenyl) thiazole (also known as SK1 II) has been shown to induce SPHK1 degradation through lysosomal pathway (Ren et al., 2010). Chloroquine (CQ), on the other hand, has been shown to reverse SK1 II mediated degradation of SPHK1 (Ren et al., 2010). CQ though does not affect *de novo* synthesis of S1P derivatives; it restores S1P level in the cell through disrupting autophagy or lyso-somal degradation of enzymes by increasing the lysomal pH. Therefore, investigation on whether SK1 II also has its effect in RBC/parasite infected RBC which lack lysosome, or whether CQ modulates SPHK1 function in these cells upon SK1 II treatment would be important for possible targeted degradation of SPHK1.

Stimulation of S1P degradation in parasite infected RBC would be another appreciable approach. Although, RBC was reported as incapable of degrading S1P due to lack of S1PL and SPP enzymes (Ito et al., 2007), low level of these enzymes have been detected (Selim et al., 2011). Further, marked reduction of S1P in prolong storage of RBC (Selim et al., 2011; Dong et al., 2012) support the plausible S1P degradation in RBC. It is unknown whether expression of these S1P degrading enzymes are induced or affected by the malaria parasite. However, SPP induction in iRBCs would be helpful in two ways. First, S1P level in RBCs would be reduced as required for therapeutics because of its degradation into Sphingosine and phosphate. Second, increased intra-cellular content of sphingosine might play an additional role of inducing eryptosis due to phosphatidyl serine exposure at RBC surface (erythrocyte death characterized by cell shrinkage) as reported previously (Qadri et al., 2011). However, it is unknown whether sphingosine could induce eryptosis in iRBCs as well. Since RBC serves as important source for the plasma level of S1P, cellular depletion of S1P via facilitated import into plasma through chaperone proteins (especially through ApoM-HDL) would be of therapeutic interest. This perspective of intra-cellular S1P depletion would be valuable by affecting parasite growth in infected RBCs as well as increasing plasma level of S1P as required for protection. Although albumin too function as S1P acceptor, the observation of increased albumin in cerebrospinal fluid impairing BBB function of Malawian children with CM (Brown et al., 2001) and reported S1PR_1_ internalization upon albumin-S1P binding precludes its utility in therapeutics use in malaria. Of note, administration of engineered ApoM with long plasma half-life has resulted into increased plasma concentration of S1P and cardiac protection with recovery from stroke in experimental mice (Swendeman et al., 2017). Similar study is warranted in experimental model of malaria and whether there is deprivation of the RBC content of S1P through increased efflux upon adequate bio-availability of engineered ApoM needs to be investigated. Because, parasite dependency on RBC content of S1P for survival in infected cell may complicate its export to plasma. Alternatively, induction of S1P transporters in RBC and platelets would be additional opportunity for S1P-targeted drug development in malaria.

## Conclusion and Future Perspectives

In summary, S1P seems to play a double-edged sword in malaria pathogenesis. While intracellular content of S1P in infected RBCs is essential for parasite growth and development, its low level in plasma is associated with cerebral malaria in mouse model and human studies. However, how reduced S1P concentration in plasma affects other forms of sub-clinical severe malaria is yet to be materialized. Although diminished S1P concentration in plasma is likely to induce S1P synthesis in RBC, it is yet to be confirmed in parasite infected and/or non-infected RBCs through experimental studies in malaria. Further, whether S1P is involved in inducing rosette formation during *falciparum* infection would be important to understand rosette mediated complicacy in severe malaria. The fact that bioavailability of S1P is a tightly regulated process and its function is modulated by acceptor chaperones in the plasma. Thus, screening of functional variants in genes regulating S1P level in the plasma and S1P receptors, proportion of plasma S1P bound to albumin or ApoM HDL and their association with clinical manifestation of malaria would be helpful. Although kidney function to prevent urinal secretion of S1P, in malaria related renal failure (the most common severe complication after cerebral malaria), urinal detection of S1P could serve as a prognostic indicator and needs investigation in this line. Hitherto, substantial evidences suggest plasma S1P is crucial for differential manifestation of malaria. Therefore, selective inhibition of S1P synthesis in RBC, its degradation or abatement though supplementation of adequate acceptor molecules leading to augmented plasma level S1P may impair RBC growth of parasite and protect from severe malaria. Although the involvement of S1P in malaria pathogenesis has been studied only recently; more studies are warranted to answer several of these un-resolved questions before its implication as therapeutics.

## Author Contributions

GD: conceived the original idea, prepared all figures, and the first draft of manuscript. SS: interpreted the manuscript drafting for important intellectual content. All authors discussed and contributed to the final manuscript.

## Conflict of Interest

The authors declare that the research was conducted in the absence of any commercial or financial relationships that could be construed as a potential conflict of interest.
